# Nematicidal, Fungicidal and Bactericidal Activities of Manganese (II) Complexes with Heterocyclic Sulphonamide Imines

**DOI:** 10.1155/MBD.2002.53

**Published:** 2002

**Authors:** Mukta Jain, Sampat Nehra, P. C. Trivedi, R. V. Singh

**Affiliations:** 1 Department of Chemistry University of Rajasthan Jaipur 302 004 India; 2 Department of Botany University of Rajasthan Jaipur 302 004 India

## Abstract

Some manganese(II) complexes derived from different sulphadrugs and heterocyclic ketones have been prepared. These complexes have been characterized on the basis of elemental analyses, molecular weight determinations, conductivity measurements, infrared, ESR and magnetic measurements. The spectral data suggest that the ligands act in a monobasic, bidentate manner coordinating through nitrogen atom. A high spin tetrahedral geometry around this metal has been proposed on the basis of magnetic and spectral studies. The isolated products are coloured solids, soluble in DMSO, DMF and MeOH. All the complexes are monomeric in nature as indicated by their molecular weight determinations and conductivity measurements in dry DMF show them to be non-electrolytes. All the ligands and their corresponding complexes have been screened for their fungicidal, bactericidal and nematicidal activities.


### Nematicidal, Fungicidal and Bactericidal Activities of Manganese (II) Complexes With Heterocyclic Sulphonamide Imines

Mukta Jain[1], Sampat Nehra[2], P.C. Trivedi[2] and R.V. Singh[1*]

[1]*Department of Chemistry,* [2]*Department of Botany,*
*University of Rajasthan, Jaipur - 302 004, India*
*E-mail : kudiwal@datainfosys.net; Fax: +91-141-519221*

### ABSTRACT

Some manganese(II) complexes derived from different sulphadrugs and heterocyclic ketones have been prepared. These complexes have been characterized on the basis of elemental analyses, molecular weight determinations, conductivity measurements, infrared, ESR and magnetic measurements. The spectral data suggest that the ligands act in a monobasic, bidentate manner coordinating through nitrogen atom. A high spin tetrahedral geometry around this metal has been proposed on the basis of magnetic and spectral studies. The isolated products are coloured solids, soluble in DMSO, DMF and MeOH. All the complexes are monomeric in nature as indicated by their molecular weight determinations and conductivity measurements in dry DMF show them to be non-electrolytes. All the ligands and their corresponding complexes have been screened for their fungicidal, bactericidal and nematicidal activities.

### INTRODUCTION

The applications of silver(I), zinc(II) and cerium(III) compounds of sulphadiazine in toxic burn therapy have led to renewed interest in studying the metal complexes of the sulphadrugs /1/. Sulphisoxazole and 4-amino-N-(3,4-dimethyl-5-isoxazolyl) benzenesulphonamide are used to treat urinary tract infections /2/. Many chemotherapeutically important sulpha drugs, like sulphadiazine, sulphathiazole and sulphamerazine, possess $SO_2NH$ moiety as an important toxophoric function. 5-Methyl-2-sulphanilamide-1,3,4-oxadiazole has highly specific *in vitro* activity against *Mycobacterium tubercolasis* /3/.

Manganese toxicity is a serious health hazard with severe pathologies of the central nervous system /4/. Some of the major biological roles of Mn-bound enzymes and proteins are generation of $O_2$ in photosynthesis, essentially involving splitting of $H_2O$, and destroying the superoxide ion radical (by the Mn enzyme super oxide dismutase) formed as a product of oxidation by $O_2$ in biological systems /4/. Manganese enzymes are also involved in diverse metabolic pathways including DNA synthesis, sugar metabolism and protein modification, to mention a slow[4]. In order to develop new antifungal compounds of high potency, these two biologically important moieties were coupled and with the resulting molecules, metal chelates were synthesized. The complexing behaviour of sulphonamide imines towards many metals has been studied extensively, but no work has appeared on manganese(II) complexes. Therefore, it was considered of interest to synthesize and characterize such complexes by reacting manganese chloride and acetate with several sulphonamide imines derived in the following way :

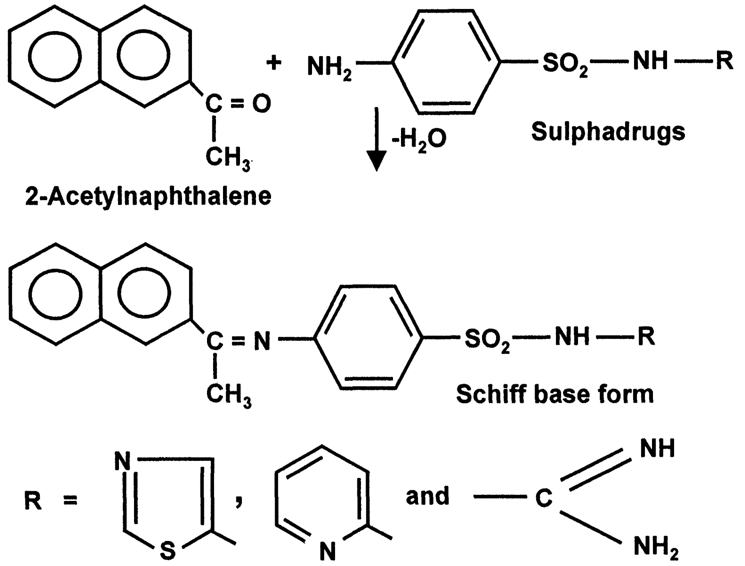

### EXPERIMENTAL

All the chemicals and solvents used were dried and purified by standard methods before use.

### Preparation of Biologically Active Imines

The ligands were prepared by the condensation of 2-acetylnaphthalene with sulphathiazole, sulphaguanidine, and sulphapyridine in 1:1 molar ratio in alcohol. The reaction mixture was refluxed in ethanolic medium (50mL) for about 5-6 hours in a water bath. On cooling, crystals of the imines separated out which were washed with ethanol, dried and recrystallized with acetone and dried *in vacuo*. These were characterized and analysed before use.

1. 2-Acetylnaphthalenesulphathiazole, (2-Ac-N-StH), white solid, M.P. 165-167°C
2. 2-Acetylnaphthalenesulphapyridine, (2-Ac-N-SpH), white solid, M.P. 162-164°C
3. 2-Acetylnaphthalenesulphaguanidine, (2-Ac-N-SgH), white solid, M.P. 140-142°C

### Synthesis of Manganese (II) Complexes

Equimolar and biomolar reactions of manganese chloride and acetate with the imines were carried out in anhydrous methanol. The mixture was refluxed for several hours by column method and then cooled to room temperature. The solvent was removed, residue was dried *in vacuo* after being repeatedly washed with dry cyclohexane and finally the complexes were recrystallized in methanol. The important properties and physical data of the complexes are reported in Table-1.

**Table 1**

Physical Properties of Manganese Complexes

| Compound | Colour | M.P. (°C) | Yield (%) | Mn Found (Calcd.) | N Found (Calcd.) | S Found (Calcd.) | Mol. Wt. Found (Calcd.) |
|---|---|---|---|---|---|---|---|
| $Mn(CH_3COO)(2\text{-}Ac\text{-}N\text{-}St)H_2O$ | Off white | 162-164 | 88 | 9.86 (10.20) | 7.54 (7.80) | 11.80 (11.90) | 502.42 (538.48) |
| $[Mn(2\text{-}Ac\text{-}N\text{-}St)_2]$ | Pitch | 170-172°C | 82 | 6.21 (6.32) | 9.18 (9.68) | 14.46 (14.77) | 841.92 (867.91) |
| $[Mn(CH_3COO)(2\text{-}Ac\text{-}N\text{-}Sg)H_2O]$ | Light blakish | 230-232°C | 79 | 10.70 (11.04) | 10.82 (11.2) | 6.13 (6.44) | 460.21 (497.38) |
| $[Mn(2\text{-}Ac\text{-}N\text{-}Sg)_2]$ | Light brown | 242-244 | 82 | 6.54 (6.99) | 14.00 (14.26) | 7.82 (8.16) | 760.29 (785.71) |
| $[Mn(CH_3COO)(2\text{-}Ac\text{-}N\text{-}Sp)H_2O]$ | Brown | 142-144°C | 85 | 9.91 (10.31) | 7.41 (7.89) | 5.81 (6.02) | 500.48 (532.48) |
| $[Mn(2\text{-}Ac\text{-}N\text{-}Sp)_2]$ | Brown | 102-104°C | 88 | 6.24 (6.41) | 9.56 (9.81) | 7.02 (7.49) | 818.94 (855.91) |
| $[MnCl(2\text{-}Ac\text{-}N\text{-}St)H_2O]$ | Off white | 150-151°C | 83 | 10.56 (10.66) | 7.69 (8.16) | 11.98 (12.45) | 482.21 (514.89) |
| $[MnCl(2\text{-}Ac\text{-}N\text{-}Sg)H_2O$ | White | 156-158 | 82 | 11.19 (11.59) | 11.39 (11.8) | 6.43 (6.76) | 441.91 (473.79) |
| $[MnCl(2\text{-}Ac\text{-}N\text{-}Sp)H_2O]$ | Off white | 154-155 | 81 | 10.54 (10.79) | 8.09 (8.26) | 6.02 (6.30) | 478.62 (508.89) |

The elemental analyses were carried out by the usual method. Manganese was estimated by EDTA titration method. The molecular weights were determined in methanol by the Ebullioscopic method. The IR spectra were recorded as KBr pellets on a Perkin-Elmer 577 grating spectrophotometer. Nitrogen and sulphur were estimated by Kjeldahl's and Messenger's methods, respectively.

### Antibacterial Activity

The bactericidal activity was evaluated by the paper-disc method /5/. The nutrient agar medium (peptone, beef extract, NaCl and agar-agar and 5mm diameter paper disc of Whatman No.1 were used. The compounds were dissolved in methanol in 500 and 1000 ppm concentrations. The filter paper discs were soaked in different solutions of the petri plates already seeded with the test organisms. The plates were incubated for 24-30 hours at $28 \pm 2^{o}C$ and the inhibition zone around each disc was measured.

### Antifungal Activity

The fungi were grown in agar medium (glucose 20g, starch 20g, agar-agar 20g and 1000 mL water) at $28 \pm 2^{o}C$ and the compounds after being dissolved in 50, 100 and 200ppm concentrations in methanol were mixed in the medium. The linear growth of the fungus was obtained by measuring the diameter of colony in petri plates after 4 days and the percentage inhibition was calculated by the formula

$$\% \text{ inhibition} = \frac{(C - T) \times 100}{C}$$

where C and T are the diameters of the fungus colony in control and test plate, respectively.

### RESULTS AND DISCUSSION

The reactions of equimolar and bimolar hydrated manganese(II) chloride and acetate with the biologically active sulphonamide imines resulted in the isolation of coloured products soluble in most of the common organic solvents and non-electrolytes in nature. The molecular weight determinations of the complexes reveal their monomeric nature which in turn indicate the tetra coordinated state for the central manganese atom. These reactions may be represented by the following equations:

$$[Mn\,(CH_3COO)_2.4H_2O] + \overset{\frown}{N\ NH} \longrightarrow [Mn\,(CH_3COO)\,(\overset{\frown}{N\ N})H_2O] + CH_3COOH + 3H_2O$$

$$[Mn\,(CH_3COO)_2.4H_2O] + 2\,\overset{\frown}{N\ NH} \longrightarrow [Mn\,(\overset{\frown}{N\ N})H_2O] + 2CH_3COOH + 4H_2O$$

$$[MnCl_2 4H_2O] + \overset{\frown}{N\ NH} \longrightarrow [MnCl\,(\overset{\frown}{N\ N})H_2O] + HCl + 3H_2O$$

$$[MnCl_2.4H_2O] + 2\,\overset{\frown}{N\ NH} \longrightarrow [Mn\,(\overset{\frown}{N\ N})_2] + 2HCl + 4H_2O$$

The reactions are quite facile and the yields are almost quantitative.

### I.R. Spectra

In the IR spectra of the free ligands the -NH stretching and deformation bands appear at 3370-3200 and 1670-1705 $cm^{-1}$, respectively. However in the spectra of manganese(II) complexes, bands due to NH

vibrations disappear, indicating deprotonation of the ligands and chelation of nitrogen with the manganese atom. In the spectra of 1:1 metal complexes, a new band also appears in the region 676-690 $cm^{-1}$, which is due to the coordinated water molecule /6/. This is not observed in the corresponding 1:2 complexes. Further, a broad band around 3400 $cm^{-1}$ may be due to νOH of water molecule /6/. The coordination of ligands through azomethine nitrogen receives further support from the appearance of new bands of medium to weak intensity in the region 410-370 $cm^{-1}$, attributable to ν M ← N vibrations /7/.

### Electron Spin Resonance Spectra

The ESR spectral studies of 1:1 and 1:2 Mn(II) complexes of 2-acetylnaphthalene with different sulphadrugs at room temperature show only one isotropic signal centered at 2.145 g, which once again suggests a four coordinated geometry for these complexes.

Thus on the basis of the above evidences a tetrahedral geometry has been proposed for 1:1 and 1:2 manganese complexes of sulpha drugs.

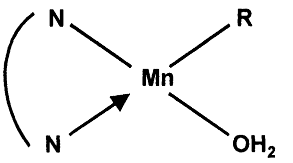

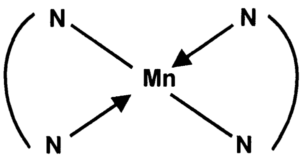

### Magnetic Susceptibility

The magnetic susceptibility of manganese(II) complexes has been determined by Gouy's method. The magnetic moment values of Mn(II) complexes have been found to be 5.9 $\pm$ 0.1 B.M, which suggests a high spin state for these complexes with a tetrahedral geometry /8/.

### Electronic Spectra

Manganese complexes show a maximum absorption band at ca. 420 nm and a charge transfer band at ca. 270nm indicating a tetrahederal geometry /9/. For these complexes in tetrahedral fields, the transitions are spin forbidden and are no longer parity forbidden. Thus the tetrahedral compounds are somewhat more intensely coloured /7/.

### Antifungal and Antibacterial Activities

It is clear from the biological screening data given in Table 2 that all of the manganese(II) complexes are more toxic than their parent conjugated bases. The increase in the activity of manganese complexes may be due to the effect of central ion in the normal cell process. The antimicrobial activity of these bases and their

**Table 2**

Biological Activity Index

| Compound | Fungicidal% inhibition *Aspergillus niger* | | | Bactericidal in (diameter) *E.coli* | | Nematicidal *Meloidogyne Hatching* (100ppm) |
|---|---|---|---|---|---|---|
| | **50** | **100** | **200** | **500** | **1000** | |
| [2-Ac-N-StH] | 47 | 66 | 79 | 7.0 | 9.8 | 13.1 |
| [$Mn(CH_3COO)$(2-Ac-N-St)$H_2O$] | 56 | 75 | 84 | 9.1 | 10.9 | 12.0 |
| [MnCl(2-Ac-N-St)$H_2O$] | 50 | 71 | 80 | 8.5 | 10.4 | 12.5 |
| [Mn(2-Ac-N-St)$_2$] | 63 | 82 | 91 | 10.3 | 12.1 | 10.5 |
| [2-Ac-N-SpH] | 45 | 63 | 74 | 6.1 | 8.6 | 14.2 |
| [$Mn(CH_3COO)$(2-Ac-N-Sp)$H_2O$] | 52 | 71 | 80 | 8.1 | 10.1 | 13.8 |
| [MnCl(2-Ac-N-Sp)$H_2O$] | 47 | 70 | 78 | 8.0 | 9.5 | 13.9 |
| [Mn(2-Ac-N-Sp)$_2$] | 61 | 65 | 87 | 9.1 | 11.6 | 13.0 |
| [2-Ac-N-SgH] | 44 | 69 | 72 | 5.8 | 8.7 | 15.0 |
| [$Mn(CH_3COO)$(2-Ac-N-Sg)$H_2O$] | 52 | 64 | 81 | 7.9 | 10.0 | 14.4 |
| [MnCl(2-Ac-N-Sg)$H_2O$] | 45 | 64 | 76 | 7.5 | 9.2 | 14.7 |
| [Mn(2-Ac-N-Sg)$_2$] | 58 | 75 | 86 | 9.0 | 11.2 | 13.4 |

complexes can be ascribed to hydrogen bond formation between the nitrogen (>C=N) atom of the compounds and some bioreceptors in the cells of the microorganism, which in turn block the synthesis of proteins by inhibiting the movement of ribosome along with RNA /10/. This inhibits the synthesis of DNA in the cell nucleus. The greater toxicity of the complexes than the bases can also be explained by the greater lipophilic character of the complexes /11/. It has also been observed that the toxicity of conjugated bases and complexes decreases on lowering the concentration.

Chelation reduces the polarity of the metal ion mainly because of partial sharing of its positive charge with the donor groups and possible $\pi$-electron-delocalisation over the whole chelate ring.

### Nematicidal Activities

Phytonematodes occur throughout the world, infect all major and minor crop plants and cause substantial reductions in crop yield and quality of produce /12/. Estimated overall average yield /13/ loss of the world's major crops due to damage by plant parasitic nematodes is 12.3%. It is estimated that 50% crop losses are caused by all kinds of pests together and nematodes' share in this may be about 5%. The nematode population levels present in soil are directly correlated with damage to cereal crops /14/. In India overall crop losses due to nematodes have been estimated /15/ as 10.6%.

In India plant parasitic nematodes *Meloidogyne incognita* and *Meloidogyne javanica* are the most abundant in the plains /16/. Nematode *Meloidogyne incognita* is known to attack more than 3000 host plants /17/. *Meloidogyne incognita* produce galls on the roots of many host plants and are responsible for 44.87

percent yield loss in brinjal /18/.

The literature of past work concerning nematode problems has indicated that there is an urgent need to check this pest by control practices, using various chemicals. For the experiment, egg masses were separated from heavily infected brinjal roots and washed under running water. For obtaining the pure quantities of *Meloidogyne incognita* eggs a step by step procedure was adopted, viz. cutting the clean root, addition of 1% NaOCl solution, shaking it and than sieving through 150 and 400 mesh sieves /19/. For each chemical 230 eggs of this nematode were counted and replicated three times. At this experiment, temperature range was 30 $\pm$ 2°C. The eggs were treated with complexes dissolved in 100ppm for 24 hrs. for each treatment. The observations in relation to hatching of these *Meloidogyne* eggs were noted. Results revealed that maximum hatching was recorded in control ($H_2O$) treatment but in eggs treated with different chemicals, very poor hatching was observed, hence nematicidal properties were recorded.

### ACKNOWLEDGEMENT

The authors are thankful to University Grants Commission, New Delhi, India for financial assistance.

### REFERENCES

1 Wilson and Gisvold's *Textbook of Organic Medicine and Pharmaceutical Chemistry*, 8th Edn., R.F. Deorge (Ed.), JB Lippinccott, Philadelphia, 1982; p. 162.
2 G. Kanagaraj and G.N. Rao, *Indian J. Chem.*, **32A**, 594 (1993).
3 H. Singh, V.K. Srivastava, S.N. Shukla, M.K. Srivastava and M.K. Upadhyay, *Indian J. Chem.*, **33A**, 350 (1994).
4 A. Sigel and H. Sigel, *Indian J. Chem.*, **39A**, 1340 (2000).
5 N.C. Bhardwaj and R.V. Singh, *Indian J. Chem.*, **33A**, 423 (1994).
6 N. Kanoongo, R.V. Singh and J.P. Tandon, *Synth. React. Inorg. Met.-Org. Chem.*, **19**, 113 (1989).
7 A. Horriman, *Coord. Chem. Rev.*, **28**, 147 (1979).
8 H.B. Singh, S. Maheshwari and N. Wasi, *Synth. React. Inorg. Met.-Org. Chem.*, **15**, 335 (1985).
9 P.P. Bhargava, R. Bembi and M. Tyagi, *J. Indian Chem. Soc.* **60**, 214 (1983).
10 J.G. Horsfall, *Bot. Rev.*, **11**, 357 (1945).
11 R.S. Srivastava, *Inorg. Chim. Acta*, **56**, 65 (1981).
12 S. Nehra, Integrated Management of Root-Knot Nematode, *Meloidogyne incognita* associated with Ginger, Ph.D. Thesis, Univ. of Raj. Jaipur (2001).
13 J.N. Sasser and D.W. Freckman, in: *Vistas on Nematology*, J.A. Veech and D.W. Dickson (Eds.), Society of Nematologists, Inc., Hyatsville, Maryland, 1987; p. 7.
14 K. Singh and G. Swaroop, Studies on the population of *Heterodera avenae* causing Molya Disease of wheat and barley in Rajasthan. *Indian Phytopath*, **17**, 212 (1964).

15 A.R. Seshadri, Nematology in India. Achievement and Prospects. In: *Plant Parasitic Nematodes of India Problems and Progress*, G. Swaroop and D.R. Dasgupta (Eds.), IARI, New Delhi, 1986; p.497.

16 S.B. Chattopadhyay and S.K. Sengupta, Root-knot disease of jute in West Bengal, *Univ. Sci.*, **2418**, 276 (1955).

17 P. Parvatha Reddy and R.M. Khan, Integrated management of root-knot nematode infecting okra, **2**, 115 (1991).

18 K. Krishnappa, K.G.H. Setty and K.S. Krishna Prasad, Crop losses assessment in Brinjal due to root-knot nematode, *Meloidogyne Oncognita. Nematol. Soc. India Symp.,* Coimbatore, 1981.

19 M.A. Mc. Clure, T.H. Kruk and L. Misaghi, A method for obtaining quantities of clean *Meloidogyne* eggs. *J. Nematol.,* **5**, 230 (1973).

